# Facile Synthesis of Pd-Ir Nanocubes for Biosensing

**DOI:** 10.3389/fchem.2021.775220

**Published:** 2021-11-24

**Authors:** Jiuxing Li, Yingfu Li

**Affiliations:** Department of Biochemistry and Biomedical Sciences, Michael G. DeGroote Institute of Infectious Disease Research (IIDR), McMaster University, Hamilton, ON, Canada

**Keywords:** Pd-Ir nanocubes, peroxidase-mimicking nanozymes, immunoassay, aptamer, fluorescent colorimetric biosensors, PSA, RNase HII

## Abstract

Displaying extremely high peroxidase-like activity and uniform cubic structure enclosed by (100) facets, Pd-Ir nanocubes are an attractive nanomaterial for bioanalysis. However, there exists a great challenge to deposit atomic layers of Ir on the surface of Pd nanocubes due to the relatively low energy barrier of homogeneous nucleation of Ir atoms compared to heterogeneous nucleation. Here, a simple and surfactant-free approach is presented to synthesize Pd-Ir nanocubes with atomic Ir shell thickness in an aqueous solution at room temperature. Biomolecules such as antibodies and nucleic acids have free access to the surface of Pd-Ir nanocubes. Applications of Pd-Ir nanocubes in immunoassays and aptamer-based biosensors are realized, exploiting the excellent peroxidase activity and fluorescence quenching ability of Pd-Ir nanocubes. This work makes a significant step forward towards the practical utility of Pd-Ir nanocubes in bioanalysis.

## Introduction

In recent years, nanomaterials have attracted increasing research interests in bioanalysis due to their outstanding optical, electronic, and catalytic properties (Link and El-Sayed, 1999; Burda et al., 2005; Willets and Van Duyne, 2007; Anker et al., 2008; Saha et al., 2012; Wei and Wang, 2013; Lin et al., 2014; Wu et al., 2019). Nanomaterials have been widely explored to achieve enzyme-like catalysis, electrocatalysis, specific adsorption of nucleic acids, efficient fluorescence quenching, creation of nanostructured electrodes, among other bioanalytical applications (Lu et al., 2009; He S. et al., 2010; Loh et al., 2010; Wang et al., 2010; Wang et al., 2011; Wei and Wang, 2013; Zhu et al., 2013; Lin et al., 2014; Tan et al., 2015; Peng et al., 2017; Wu et al., 2019). Peroxidase-mimicking nanozymes including metal oxides [Fe_3_O_4_ (Gao et al., 2007; Wei and Wang, 2008), Co_3_O_4_ (Mu et al., 2012), V_2_O_5_ (André et al., 2011), and CuO (Chen et al., 2011)], carbon materials (Song et al., 2010a; Song et al., 2010b), and noble metal nanomaterials (Jia et al., 2002; He W. et al., 2010; Jv et al., 2010; He et al., 2011; Xia et al., 2014), have been employed as alternatives to horseradish peroxidase (HRP) for antibody conjugation and signal output in immunoassays, due to their easy synthesis and increased stability (Wei and Wang, 2013; Wu et al., 2019). However, most nanozymes are subject to shortcomings in size and morphology control, large batch-to-batch variation, and limited enhancement in catalytic activity. As for adsorption-based fluorescent bioanalysis, two-dimensional (2D) nanomaterials, such as graphene oxide (Lu et al., 2009), MoS_2_ (Zhu et al., 2013), Ta_2_NiS_5_ (Tan et al., 2015), and covalent organic framework (Peng et al., 2017) are representative nanomaterials, attributable to their fairly flat surfaces. However, these 2D nanomaterials also face problems of large batch-to-batch variations and varying performance.

Pd-Ir nanocubes have great potential to circumvent the issues encountered by nanomaterials in bioanalysis, due to their superior properties including extremely high catalytic activity, excellent stability, good batch consistency, efficient fluorescence quenching, and uniform cubic structure with flat (100) facets (Xia et al., 2014; Xia et al., 2015; Ye et al., 2017; Zhu et al., 2019a; Zhu et al., 2019b). However, it has been a great challenge to deposit atomic Ir layer on Pd nanocube surfaces, due to the relatively low energy barrier for homogeneous nucleation of Ir atoms compared to heterogeneous nucleation on Pd nanocubes (Stowell and Korgel, 2005; Migowski et al., 2010; Xia et al., 2014). Most synthesis reactions result in amorphous Ir-coated Pd nanocubes, which compromise their stability, synthesis reproducibility, and uniform structure (Liu et al., 2013; Zanata et al., 2019). Harsh conditions including high temperature, surfactants, organic solvent, and flow injection in an oil bath, are required to successfully deposit thin Ir layers on Pd nanocube surfaces (Xia et al., 2015; Ye et al., 2017; Zhu et al., 2019a; Zhu et al., 2019b). However, synthesis under such conditions are not compatible with biomolecular adsorption, not easy to scale up, not environmentally friendly, restricting various applications of Pd-Ir nanocubes in bioanalysis.

The synthesis of Pd-Ir nanocubes includes two steps: Pd nanocube synthesis and Ir deposition. Previously, we reported a simple one-pot method for Pd nanocube synthesis (Li and Li, 2021). Herein, we describe a simple and surfactant-free method for depositing atomic Ir shells on Pd nanocubes in an aqueous solution at room temperature. The Pd-Ir nanocubes show high peroxidase activity and free accessibility for biomolecules. The nanomaterials are further exploited for the development of an immunoassay to detect PSA (prostate-specific antigen) as well as fluorescent and colorimetric biosensors of *C. difficile* RNase HII.

## Experimental Section

### Chemicals and Reagents

Fluorescein-labelled DNA aptamer (FAM-ARH1t6: TTA CGT CAA GGT GTC ACT CCG CCA GGT GTG CGA CGG TCG T-FAM) were purchased from Integrated DNA Technologies (Iowa, United States) and purified by 10% denaturing PAGE (polyacrylamide gel electrophoresis). Sodium tetrachloropalladate (II) (Na_2_PdCl_4_, 98%), L-ascorbic acid (AA, ≥99%), polyvinylpyrrolidone (PVP, M.W. ≈ 55,000), potassium bromide (KBr, ≥99%), sodium hexachloroiridate (III) hydrate (Na_3_IrCl_6_ • xH_2_O, M.W. = 473.9), sodium borohydride (NaBH_4_, 98%), sodium acetate (NaOAc, ≥99%), acetic acid (HOAc, ≥99.7%), hydrogen peroxide solution (30% w/v in H_2_O), 3,3′,5,5′-tetramethylbenzidine (TMB, >99%), dimethylformamide (DMF), sulfuric acid (H_2_SO_4_, 95–98%), sodium phosphate dibasic (Na_2_HPO_4_, ≥99%), potassium phosphate monobasic (KH_2_PO_4_, ≥99%), sodium chloride (NaCl, ≥99.5%), potassium chloride (KCl, ≥99%), hydrochloric acid (HCl, 37%), horse radish peroxidase (HRP, ≥99%), human prostate specific antigen (PSA, ≥99%), bovine serum albumin (BSA, ≥98%), and Tween-20 were all obtained from Sigma–Aldrich. HRP-goat anti-mouse IgG conjugate and goat anti-mouse IgG were from Thermo Fisher Scientific, Inc. Rabbit anti-PSA polyclonal antibody (pAb) and mouse anti-PSA monoclonal antibody (mAb) were both obtained from Abcam plc. *C. difficile* RNase HII is expressed and purified in our lab using *E. coli* cells containing an expression vector for the target protein.

### Synthesis of Pd-Ir Nanocubes

Pd nanocubes with different sizes (7, 18 and 51 nm) were prepared according to our previously reported method (Li and Li, 2021). Pd-Ir nanocubes were synthesized by deposition of Ir atoms on the surface of Pd nanocubes. Briefly, Pd nanocubes (50 μl, 44 nM, 18 nm) were diluted by water (1 ml) to 2.2 nM in a centrifuge tube (1.5 ml) and centrifuged at 14,000 rpm for 10 min. After removing the supernatant, the Pd nanocubes pellets were resuspended by water (1 ml). Then, Na_3_IrCl_6_ (20 µl, 10 mM) and NaBH_4_ (20 µl, 100 mM) were added to the Pd nanocube solution sequentially and kept at room temperature for 1 h. The NaBH_4_ (20 µl, 100 mM) addition process was repeated once, and the mixture was allowed to react at room temperature for 4 h. Pd-Ir nanocubes were obtained and stored at room temperature before use. The concentration of Pd-Ir cubes synthesized by this method was 2.2 nM according to the concentration of Pd nanocubes.

### Steady-State Kinetic Assays of Peroxidase-like Activity of Pd-Ir Nanocubes

The peroxidase-like activity of Pd-Ir nanoparticles was demonstrated by catalyzing the oxidation of TMB by H_2_O_2_. Pd-Ir nanocubes (50 µl, 1.376 × 10^–13^ M) were mixed with TMB substrate (50 µl, containing 1.6 mM TMB, 4 M H_2_O_2_, 40 mM acetate buffer, pH = 4.0) in the wells of a 96-well microtiter plate. After reacting at room temperature for 20 min, the reaction was terminated by H_2_SO_4_ (20 µl, 2 M). A microplate reader was used to measure the absorbance of the TMB oxidized product at 450 nm.

A steady-state kinetic assay of Pd-Ir nanocubes for catalyzing the oxidation of TMB by H_2_O_2_ was conducted by monitoring the catalytic reaction in a real-time manner. Pd-Ir nanocubes (50 µl, 2.752 × 10^–13^ M), H_2_O_2_ (50 µl, 8 M), and TMB substrate (100 µl, 1.6 mM TMB in 40 mM acetate buffer, pH = 4.0) were mixed in a cuvette with a path length of 1 cm. The absorbance at 653 nm was measured by UV-Vis absorption spectroscopy for 300 s with intervals of 12 s. According to the extinction coefficient of TMB oxidized product at 653 nm (3.9 × 10^–4^ M^−1^ • cm^−1^), the initial reaction velocity (ν) at a specific concentration of substrate was calculated by the initial slope of UV-Vis absorption kinetic curves. The apparent kinetic parameters could be calculated by the Michaelis-Menten equation ν = V_max_ × (S)/[K_m_ + (S)], where ν is the initial reaction velocity, V_max_ is the maximal reaction velocity, (S) represents the concentration of substrate, and K_m_ represents the Michaelis constant.

### Functionalization of Pd-Ir Nanocubes With Antibodies

Pd-Ir nanocubes are functionalized with goat anti-mouse IgG by physical adsorption. Briefly, goat anti-mouse IgG (5 µl, 1 mg/ml) and mPEG-SH (10 µl, 100 µM) were added to Pd-Ir nanocubes (1 ml, 0.55 nM) followed by incubation at room temperature for 1 h. Then, BSA (200 µl, 10% w/v) was introduced to the mixture to block Pd-Ir nanocubes by incubation at room temperature for 1 h. Finally, goat anti-mouse IgG-conjugated Pd-Ir nanocubes were washed twice with BSA solution (1 ml, 1% w/v) by centrifuging at 10,000 rpm for 5 min. Finally, the goat anti-mouse IgG-conjugated Pd-Ir nanocubes were resuspended in storage buffer (1 ml, 10 mM Na_2_HPO_4_, 1.8 mM KH_2_PO_4_, 137 mM NaCl, 2.7 mM KCl, 0.05% v/v Tween-20, 10% w/v BSA) and kept at 4°C before use.

### Pd-Ir Nanocubes for Prostate-Specific Antigen Immunoassay

Rabbit anti-PSA pAb (100 µl, 5 μg/ml, in PBS) was first attached to the wells of a microtiter plate by incubation at 4°C for 12 h, followed by three washes with PBST (200 µl, 10 mM Na_2_HPO_4_, 1.8 mM KH_2_PO_4_, 137 mM NaCl, 2.7 mM KCl, 0.05% v/v Tween-20). Then, the wells were blocked with blocking buffer (200 µl, 10 mM Na_2_HPO_4_, 1.8 mM KH_2_PO_4_, 137 mM NaCl, 2.7 mM KCl, 0.05% v/v Tween-20, 2% w/v BSA) by incubation at 37°C for 1 h, followed by washing for three times. Afterwards, PSA-containing samples in antibody dilution buffer (100 µl, 10 mM Na_2_HPO_4_, 1.8 mM KH_2_PO_4_, 137 mM NaCl, 2.7 mM KCl, 0.05% v/v Tween-20, 2% w/v BSA) were added and shaken at 100 rpm at 25°C for 2 h. After removing the samples, the wells of the microtiter plate were washed three times again with PBST. Next, mouse anti-PSA mAb (100 µl, 2 μg/ml in antibody dilution buffer) was introduced to associate with PSA by shaking at 100 rpm at 25°C for 1 h. After washing the wells three times, goat anti-mouse IgG conjugated Pd-Ir nanocubes (100 µl, 0.138 nM) were added to the wells followed by shaking at 100 rpm at 25°C for 1 h. Pd-Ir nanocubes were removed by washing three times using PBST. Finally, TMB substrate (100 µl, 0.8 mM TMB, 2 M H_2_O_2_, 20 mM NaOAc/HOAc, pH = 4.0) was added to initiate the catalytic reaction by incubation at 25°C for 20 min. The reaction was terminated with H_2_SO_4_ (20 µl, 2 M). UV-Vis absorbance at 450 nm was measured and compared to a standard working curve to determine the concentration of PSA.

In comparison, HRP-goat anti-mouse IgG conjugates were used to carry out the standard ELISA. All the experimental procedures of standard ELISA are the same as that of Pd-Ir nanocube-based immunoassay except the replacement of goat anti-mouse IgG-conjugated Pd-Ir nanocubes with HRP-goat anti-mouse IgG conjugates (100 µl, 0.5 μg/ml) and the reduction of H_2_O_2_ concentration in TMB substrates to 10 mM.

### Pd-Ir Nanocube-Based Biosensor for *C. Difficile* RNase HII Detection

All fluorescence spectra were measured by a Tecan microplate reader. In a typical assay, FAM-labelled DNA aptamer (FAM-ARH1t6, 10 µl, 1 µM) was incubated with different concentrations of protein (0.5–64 nM) in HEPES buffer (100 µl) at room temperature for 30 min. Then, Pd-Ir nanoparticles (100 µl, 22 nM) were added and incubated at room temperature for 30 min, followed by fluorescence measurements (excitation: 494 nm; emission: 522 nm).

For colorimetric detection of protein, FAM-labelled DNA aptamer, protein, and Pd-Ir nanoparticles were incubated as described above. Then, Pd-Ir nanocubes were diluted by water to a final concentration of 1.376 × 10^–13^ M. Pd-Ir nanocubes (50 µl, 1.376 × 10^–13^ M) was mixed with TMB substrate (50 µl, containing 1.6 mM TMB, 4 M H_2_O_2_, 40 mM acetate buffer, pH = 4.0) in the wells of a 96-well microtiter plate. After reacting at room temperature for 20 min, the reaction was terminated by H_2_SO_4_ (20 µl, 2 M). A microplate reader was used to measure the absorbance of the TMB oxidized product at 450 nm.

### Characterizations

The UV-Vis kinetic assays were conducted by UV-Vis absorption spectroscopy (Agilent Cary 60). The UV-Vis absorption spectra and fluorescence spectra were measured by a Tecan microplate reader. SEM analyses were performed on Hitachi S-4700 FE-SEM at 20 kV. TEM images were obtained using a JEOL transmission electron microscope (JEM-2010) operating at 200 kV. HRTEM images and EDX spectra were obtained on an FEI Titan 80–300 LB microscope operating at 300 kV. High angle annular dark-field (HAADF)-STEM analyses and EDX mapping were carried out on an aberration-corrected FEI Titan 80–300 HB Cubed microscope operating at 200 kV. The pH values of different buffer solutions were measured on a benchtop pH meter (Oakton pH 700).

## Results and Discussion

### Synthesis and Characterization of Pd-Ir Nanocubes

**FIGURE 1:**
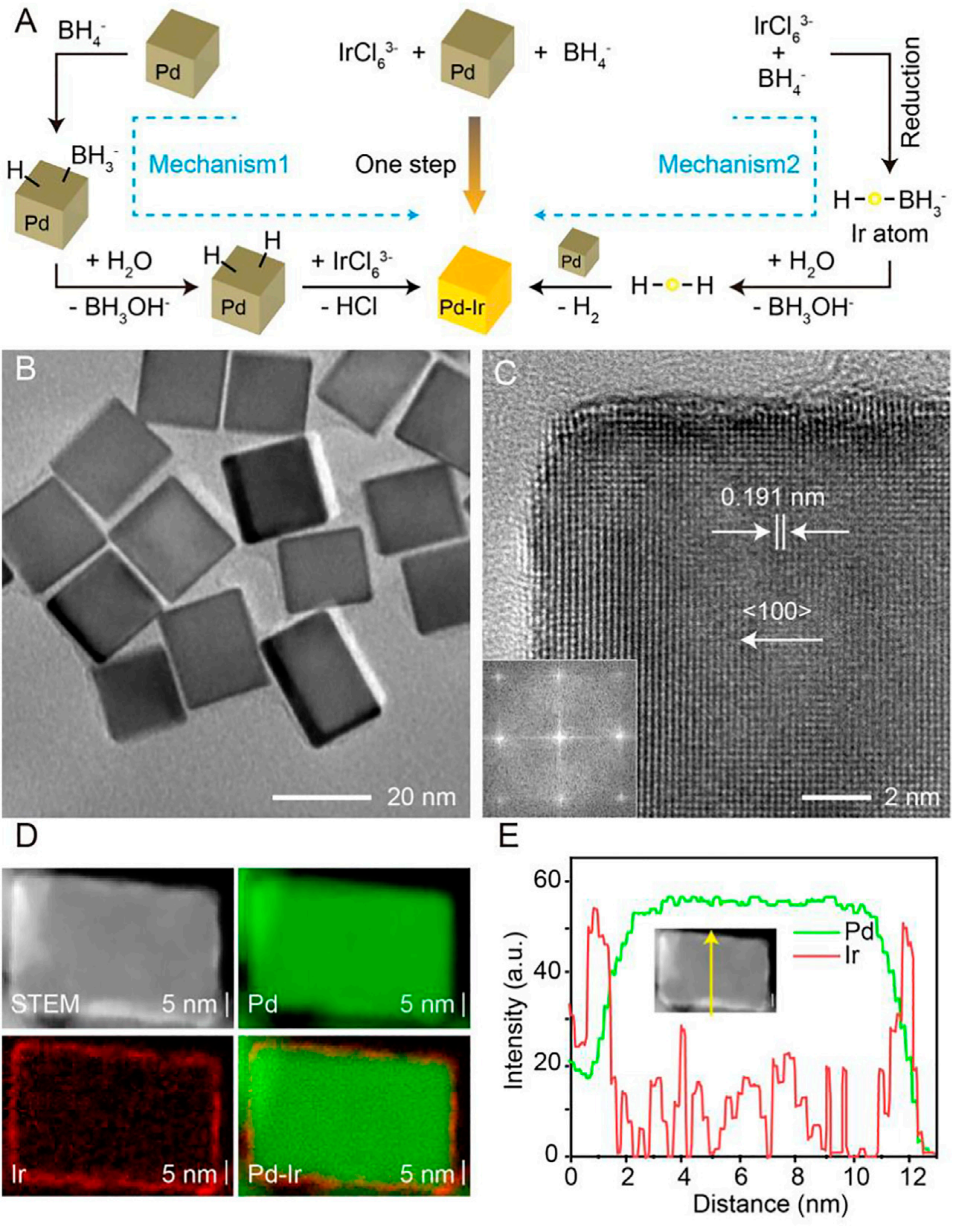
**(A)** Scheme showing the mechanism of synthesis of Pd-Ir nanocubes. **(B)** The TEM image of Pd-Ir nanocubes. **(C)** The HRTEM image (inset: corresponding Fourier transform pattern) of a corner region of a Pd-Ir nanocube. **(D)** The HAADF-STEM image and EDX maps of a Pd-Ir nanocube. **(E)** Linear EDX analysis of a Pd-Ir nanocube.

The scheme to synthesize Pd-Ir nanocubes is shown in Figure 1A. Pd nanocubes were prepared according to our previously published method (Li and Li, 2021). Then, Ir is deposited directly on Pd nanocubes through the reduction of Na_3_IrCl_6_ by NaBH_4_. Because both Pd and Ir are ideal catalysts towards the hydrolysis of NaBH_4_ for H_2_ generation (Kaufman and Sen, 1985; Muir and Yao, 2011; Retnamma et al., 2011), two mechanisms are involved in the deposition of Ir on Pd nanocubes. First, Pd nanocubes catalyze the hydrolysis of NaBH_4_ to form hydrogen-capped Pd nanocubes, which reduce IrCl_6_^3-^ to form Pd-Ir nanocubes. Second, BH_4_^−^ acts as both reducing agent and capping agent to convert IrCl_6_^3-^ into hydrogen-conjugated Ir, which deposits on Pd nanocubes with the generation of H_2_. This approach avoids the high temperature needed to assist fast diffusion of Ir (Xia et al., 2014), achieving surfactant-free Pd-Ir nanocubes.

Pd nanocubes were characterized by scanning electron microscopy (SEM, Supplementary Figure S1A) and transmission electron microscopy (TEM, Supplementary Figure S1B). A product yield higher than 98% is achieved for Pd nanocubes with a size distribution of 18.2 ± 1.8 nm (Supplementary Figure S1C). The typical high-resolution TEM (HRTEM) image of an individual Pd nanocube along the (100) zone axis clearly shows the (200) facets of face-centered cubic Pd, confirmed by the lattice spacing of 0.191 nm (Supplementary Figure S1D) (Lim et al., 2009; Xia et al., 2014; Xia et al., 2015; Zhu et al., 2019b). A thin Ir layer was deposited on the surface of Pd nanocubes by the reduction of Na_3_IrCl_6_ with NaBH_4_, achieving a smooth Ir surface (Figure 1B). The HRTEM image of a Pd-Ir nanocube (Figure 1C) along the (100) zone axis reveals continuous fringes, while the corresponding Fourier transform pattern (Figure 1C, inset) shows a square symmetry of the spots, indicating an epitaxial relationship between the single-crystal cubic Pd cores and Ir shells.

Owing to the difference of atomic number between Pd and Ir, thin Ir shells on the surface of Pd nanocubes can be resolved in the high-angle annular dark-field scanning transmission electron microscopy (HAADF-STEM) image (Supplementary Figure S1E). Statistical analysis of Pd-Ir nanocubes (Supplementary Figure S1F) demonstrates an average edge length of 19.8 ± 3.1 nm, which is 1.6 nm greater than that of Pd nanocubes, therefore signifying an average thickness of 0.8 nm Ir shells (about 3 Ir atoms) on each face of Pd nanocubes. In addition, the presence of Ir on Pd nanocubes was further verified by energy-dispersive X-ray spectroscopy (EDX) analysis (Supplementary Figure S1G), and the distribution of Ir on Pd nanocube surfaces was visualized by EDX elemental mapping (Figure 1D). The HAADF-STEM image (Figure 1D) of an individual Pd-Ir nanocube shows a cubic structure surrounded by a brighter shell, which is ascribed to the higher atomic number of Ir compared to Pd. Pd and Ir are highlighted green and red respectively. The particle core is dominated by Pd while the shell is mainly composed of a uniform Ir layer. Linear EDX scan analysis (Figure 1E) confirms the Ir shell having a thickness of about 0.8 nm. Compared to Pd nanocubes, Pd-Ir nanocubes display a lighter brown appearance (Supplementary Figure S1H) with a decrease of absorbance around 450 nm (Supplementary Figure S1H). Overall, deposition of thin Ir layers on Pd-nanocubes is achieved by a simple and surfactant-free approach, breaking through the relatively high energy barrier in the synthesis of Pd-Ir nanocubes compared to homogeneous nucleation of Ir nanoparticles.

### Optimization of Ir Deposition on Pd Nanocubes

**FIGURE 2:**
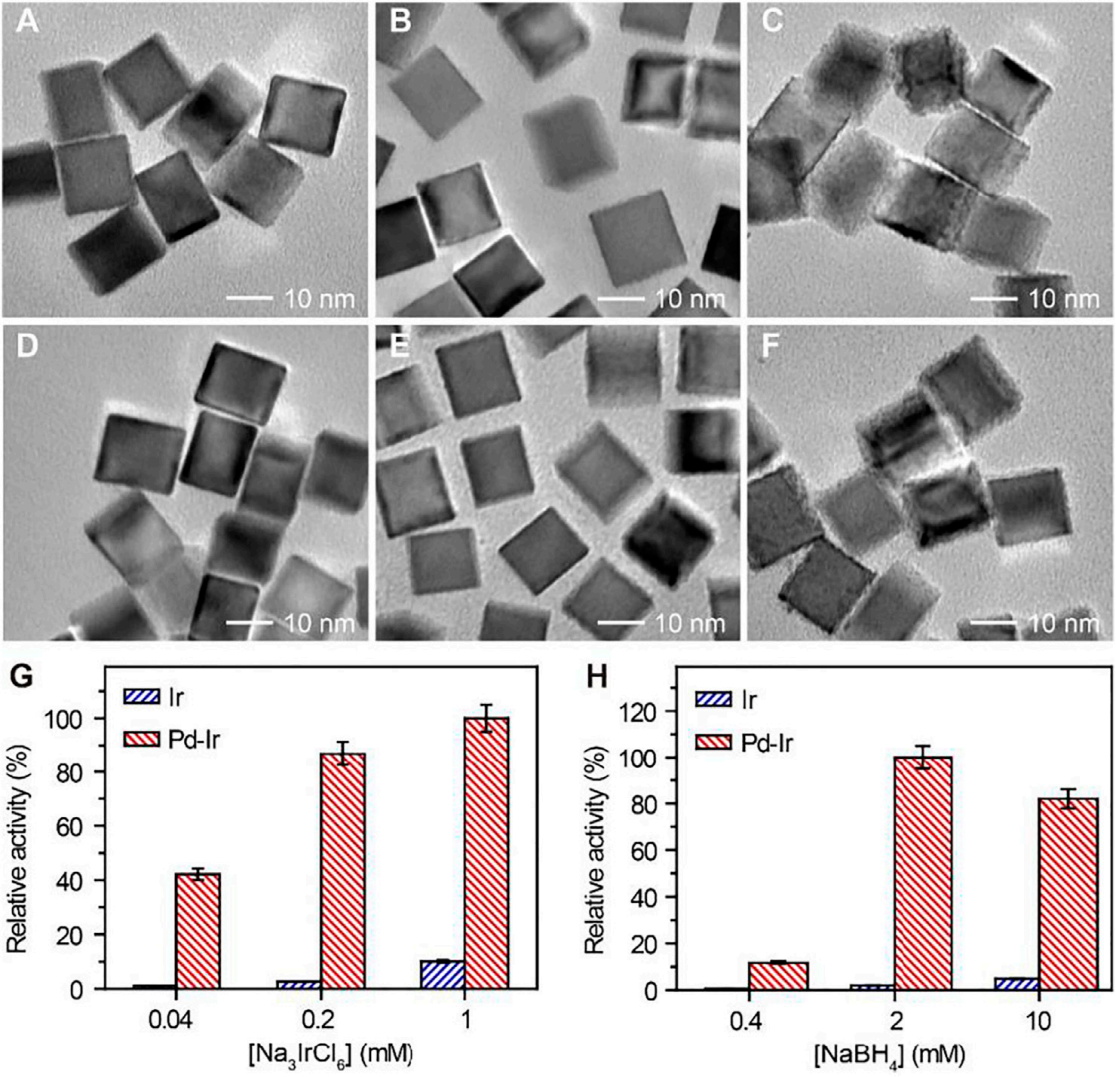
TEM images of Pd-Ir nanocubes synthesized using **(A)** 0.04, **(B)** 0.2, and **(C)** 1 mM Na_3_IrCl_6_ with 2 mM NaBH_4_. TEM images of Pd-Ir nanocubes synthesized using **(D)** 0.4, **(E)** 2, and **(F)** 10 mM NaBH_4_ with 0.2 mM Na_3_IrCl_6_. Relative peroxidase mimicking activities of Pd-Ir nanocubes synthesized with different concentrations of **(G)** Na_3_IrCl_6_ and **(H)** NaBH_4_.

To synthesize Pd-Ir nanocubes with high peroxidase activity while avoiding homogenous nucleation of Ir nanoparticles, we optimized the concentrations of Na_3_IrCl_6_ and NaBH_4_ for Ir deposition. Pd-Ir nanocubes from heterogeneous nucleation and Ir nanoparticles from homogeneous nucleation were separated by centrifugation and investigated for their peroxidase activities towards the oxidation of tetramethylbenzidine (TMB) with H_2_O_2_. As shown in Figure 2, amorphous Ir shells and significantly increased activity of Ir nanoparticles were observed when the concentrations of Na_3_IrCl_6_ were higher than 0.2 mM, or the concentration of NaBH_4_ over 2 mM. It indicates severe homogeneous nucleation happens at these conditions. To obtain Pd-Ir nanocubes with high catalytic activity and good batch consistency, the concentrations of Na_3_IrCl_6_ and NaBH_4_ were set to be 0.2 and 2 mM, respectively. This approach overcomes the difficulties in coating thin Ir layers on Pd nanocubes by controlling the reaction kinetic with Na_3_IrCl_6_ and NaBH_4_ concentrations. Moreover, the best activity was observed with the Ir deposition reaction time of 4 h (Supplementary Figure S2A) and with two additions of NaBH_4_ (Supplementary Figure S2B).

### Steady-State Kinetic Assays of Pd-Ir Nanocubes

**FIGURE 3:**
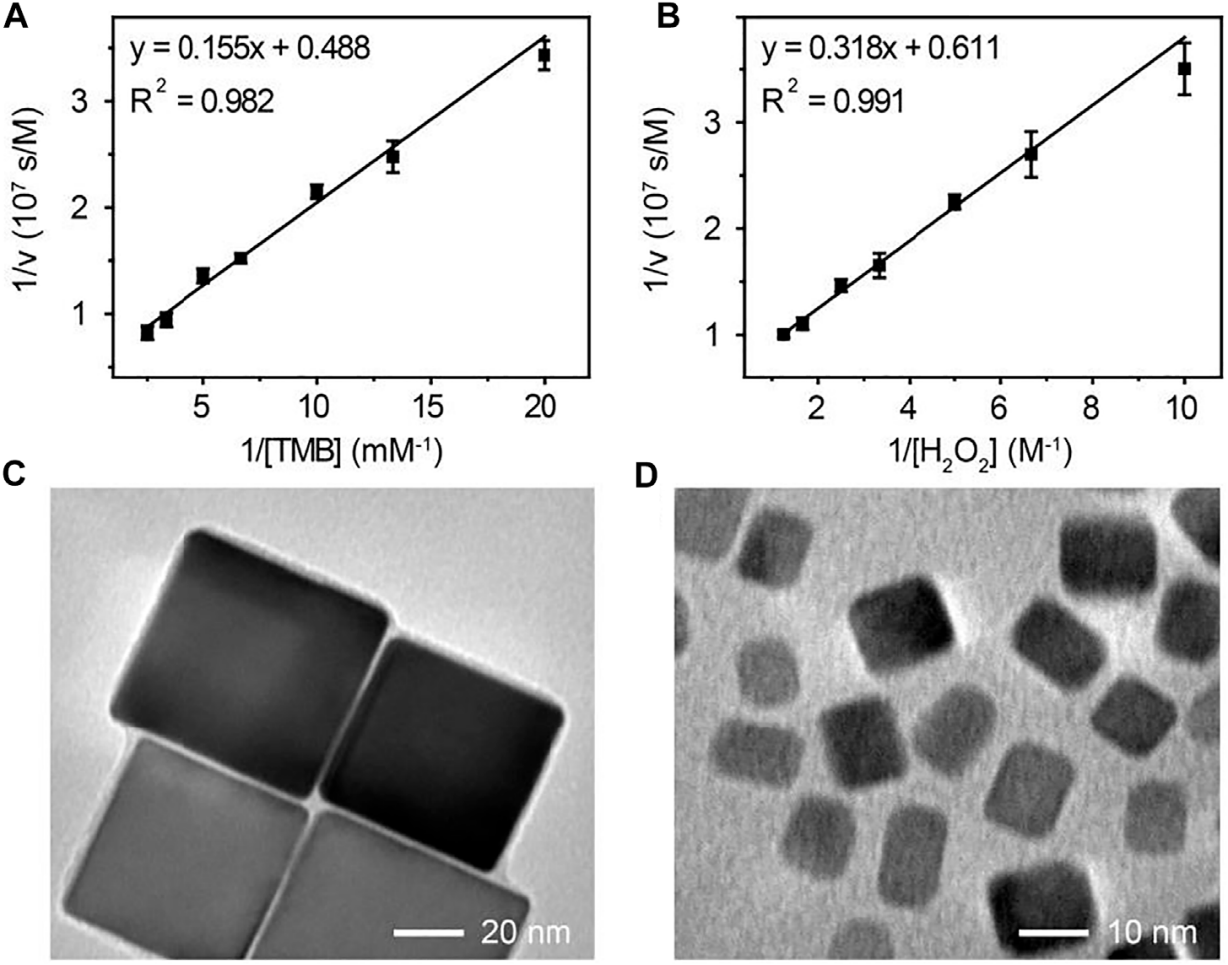
The double-reciprocal plot of initial reaction velocity against **(A)** TMB or **(B)** H_2_O_2_ concentration, the reaction catalyzed by 18 nm Pd-Ir nanocubes. The TEM images of **(C)** 51 nm and **(D)** 7 nm Pd-Ir nanocubes.

To investigate the peroxidase-like activity of Pd-Ir nanocubes, the steady-state kinetic constants for catalyzing the oxidation of TMB with H_2_O_2_ were determined using the Michaelis-Menten equation. The initial reaction velocities (ν) were plotted against the concentrations of TMB or H_2_O_2_ to obtain the Michaelis-Menten curves (Supplementary Figure S2C,D), which were then fitted to double-reciprocal plots (Figures 3A,B). The kinetic parameters of Pd-Ir nanocubes for TMB and H_2_O_2_ are listed in Supplementary Table S1. The catalytic rate constant (*k*_cat_) values of Pd-Ir nanocubes for TMB and H_2_O_2_ were higher compared to previously reported Pd-Ir nanocubes, which were about 3 orders of magnitude higher than that of HRP (Gao et al., 2007; Xia et al., 2015). The excellent peroxidase-like activity of Pd-Ir nanocubes is attributed to the intrinsic electronic shell structure of Ir, the electronic effect between Pd and Ir, and the absence of surfactants during synthesis.

**FIGURE 4:**
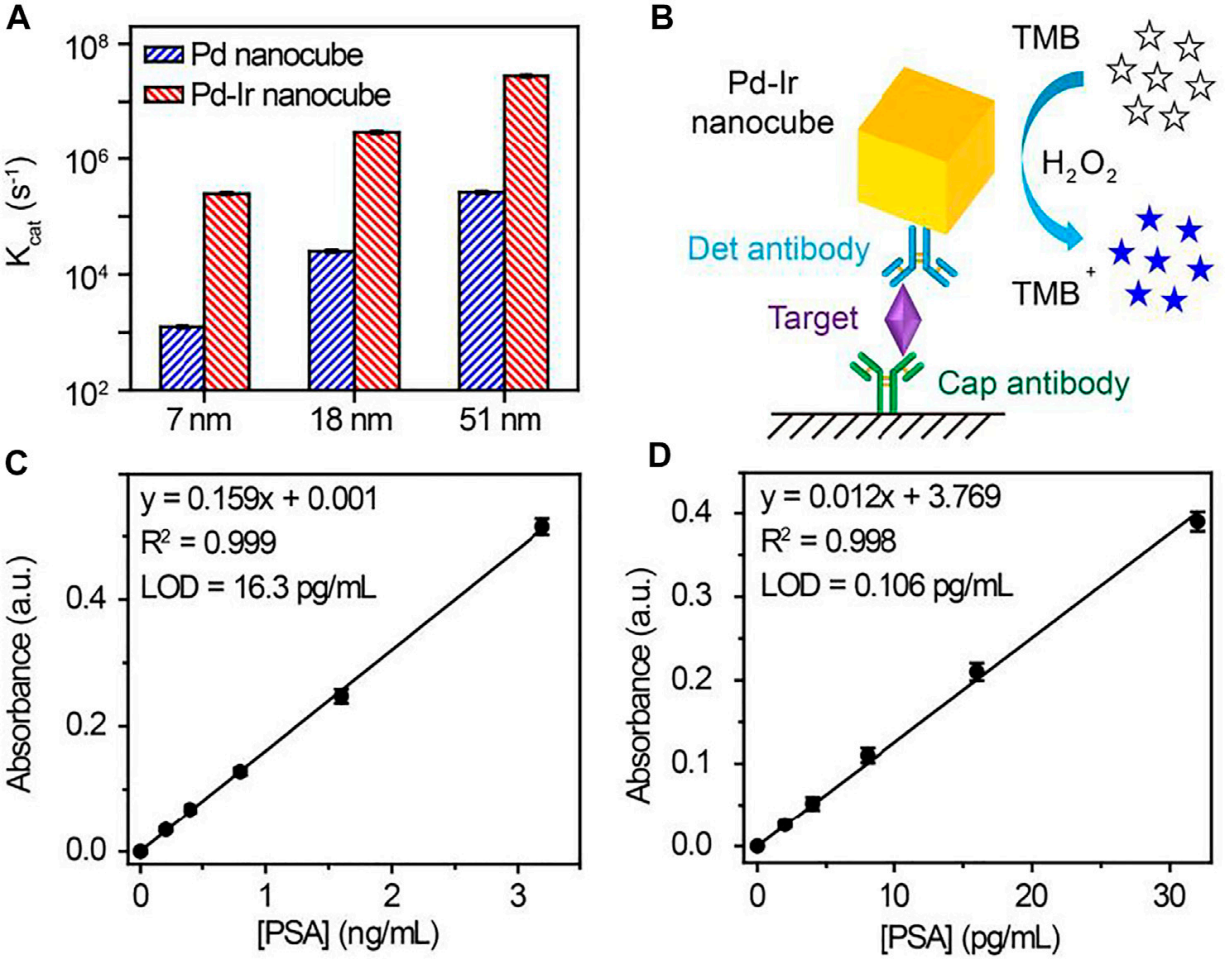
**(A)** Catalytic rate constants of Pd and Pd-Ir nanocubes with different edge lengths. **(B)** Scheme of Pd-Ir nanocube-based ELISA for the detection of PSA. Signal responses of **(C)** HRP and **(D)** Pd-Ir nanocube-based ELISA for the detection of PSA. LOD (limit of detection) is defined as the concentration of targets corresponding to a signal that is three times the standard deviation higher than zero calibrators (n = 6).

To demonstrate the versatility of this method, Pd-Ir nanocubes with different sizes were synthesized. Pd nanocubes of 51 and 7 nm were synthesized according to the literature (Li and Li, 2021). As shown in Supplementary Figure S2E,F, both Pd nanocube species show well-defined cubic shapes, indicating that they are enclosed by (100) crystal faces. Thin Ir layers were then coated on the nanoparticle surface. Pd-Ir nanocubes still maintain the cubic crystal structure with a smooth surface (Figures 3C,D), demonstrating that this approach can also be applied for the preparation of 51 and 7 nm Pd-Ir nanocubes. The catalytic rate constants of Pd and Pd-Ir nanocubes with different sizes were compared (Figure 4A). The catalytic rate constant increases with particle sizes for both Pd and Pd-Ir nanocubes, likely due to the increased surface. Pd-Ir nanocubes show about two orders of magnitude higher peroxidase activity compared to corresponding Pd nanocubes.

### An Immunoassay for Prostate-Specific Antigen

PSA, a serine protease for physiologically liquefying the seminal fluid (Lilja, 1985), has been used extensively as a cancer marker for initial diagnosis and monitoring response to treatment (Catalona et al., 1991; Barry, 2001; Wu et al., 2001; Lilja et al., 2008). Hence, PSA was chosen as the target to test the immunoassay performance of Pd-Ir nanocubes. Due to surfactant-free synthesis, Pd-Ir nanocubes can be conjugated with antibodies by physical adsorption, avoiding the time-consuming and complex process of chemical conjugation (Xia et al., 2015). As shown in Figure 4B, Pd-Ir nanocubes were used as peroxidase-mimicking enzymes and functionalized with PSA-specific antibodies for the detection of PSA. In the presence of the target, detection antibody-conjugated Pd-Ir nanocubes are immobilized on capture antibody-coated microtiter plate through the specific antibody-antigen interaction. The immobilized Pd-Ir nanocubes catalyze the oxidation of colorless TMB to blue oxidized products (TMB^+^), which turn yellow (TMB^2+^) after termination with sulfuric acid. The concentration of PSA is determined by the UV-Vis absorbance at 450 nm by comparing with a standard curve.

We first optimize the conditions to functionalize Pd-Ir nanocubes with antibodies. Bovine serum albumin (BSA) is used to block Pd-Ir nanocubes and prevent non-specific adsorption. As shown in Supplementary Figure S3A, it needed 30 min to complete the attachment of antibodies on Pd-Ir nanocubes. 10% w/v BSA and 1 h incubation at room temperature were needed to block Pd-Ir nanocubes (Supplementary Figure S3B,C). Thereafter, antibody-conjugated Pd-Ir nanocubes were used for the detection of PSA. As shown in Figures 4C,D, UV-Vis absorbance at 450 nm shows a linear relationship to the concentration of PSA for both HRP-based and Pd-Ir nanocube-based methods. The detection limits for HRP-based and Pd-Ir nanocube-based methods are 16.3 pg/ml and 0.106 pg/ml, respectively. It demonstrates that Pd-Ir nanocube-based method has two orders of magnitude higher sensitivity than the HRP-based method.

### An Aptamer-Based Dual Fluorescent and Colorimetric Biosensor for *C. Difficile* RNase HII

**FIGURE 5:**
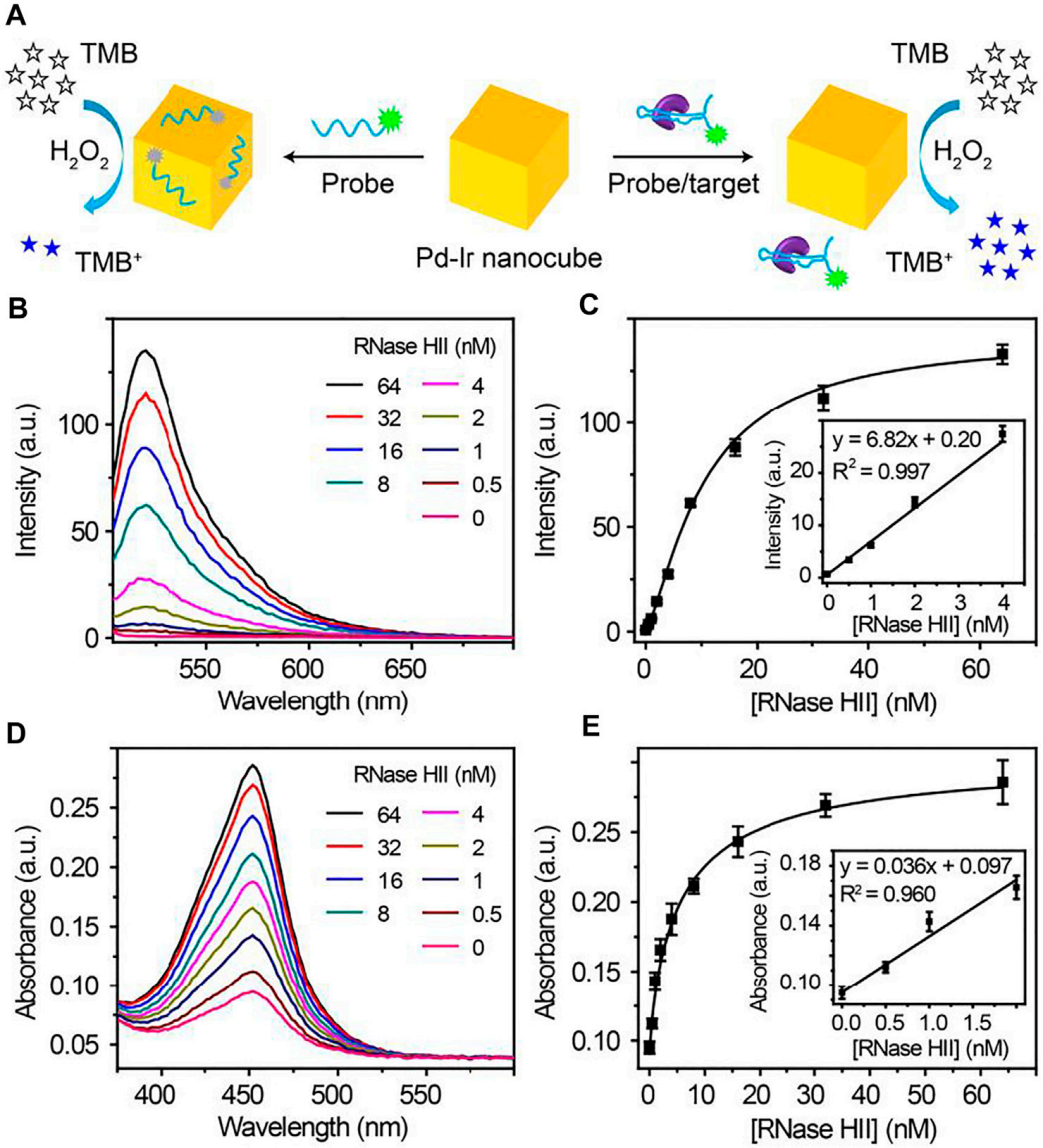
**(A)** Schematic illustration of fluorescent and colorimetric detection of *C. difficile* RNase HII using Pd-Ir nanocubes and fluorescein-labelled aptamers. **(B)** Fluorescence curves and **(C)** plot (inset: linear response) of corresponding fluorescence intensity at 522 nm towards different concentrations of *C. difficile* RNase HII; LOD = 0.15 nM. **(D)** Absorption curves and **(E)** plot (inset: linear response) of the corresponding absorbance at 450 nm towards different concentrations of *C. difficile* RNase HII; LOD = 0.29 nM. LOD (limit of detection) is defined as the concentration of targets corresponding to a signal that is three times the standard deviation higher than zero calibrators (n = 6).

Pd-Ir nanocubes were also employed for the construction of dual fluorescent and colorimetric biosensors. Because Pd-Ir nanocubes are noble metal nanoparticles with six flat uniform crystal facets, it has great potential to improve the consistency of nucleic acid adsorption, which can be employed for the design of fluorescent biosensors. However, Pd-Ir nanocubes synthesized by the traditional method were capped by surfactant, which prevents the adsorption of nucleic acids. Due to surfactant-free synthesis, Pd-Ir nanocubes prepared in this report have great potential to adsorb nucleic acids and be used for fluorescent biosensors. ARH1t6, a DNA aptamer for *C. difficile* RNase HII (Li et al., 2021), was labelled with fluorescein to investigate the nucleic acid adsorption property of Pd-Ir nanocubes. As shown in Supplementary Figure S4A, FAM-ARH1t6 can be efficiently adsorbed to the surface of Pd-Ir nanocubes synthesized in this report compared to the ones synthesized according to literature (Xia et al., 2015). FAM-ARH1t6 was then combined with Pd-Ir nanocubes and used for the detection of *C. difficile* RNase HII. The detection scheme is shown in Figure 5A. In the absence of *C. difficile* RNase HII, FAM-ARH1t6 is adsorbed to the surface of Pd-Ir nanocubes, leading to efficient fluorescence quenching of FAM-ARH1t6 and catalytic activity inhibition of Pd-Ir nanocubes. However, in the presence of *C. difficile* RNase HII, FAM-ARH1t6 switches binding partners from Pd-Ir nanocubes to RNase HII, resulting in increases of both fluorescence intensity and peroxidase catalytic activity. As shown in Figures 5B,C, the fluorescence intensity is proportional to the concentrations of *C. difficile* RNase HII, which can be quantitatively detected from 0 to 4 nM with a limit of detection (LOD) of 0.15 nM. Alternatively, *C. difficile* RNase HII can also be determined by the colorimetric method. A positive relationship is observed for the catalytic activity of Pd-Ir nanocubes with target concentrations (Figure 5D). *C. difficile* RNase HII is quantitatively detected by the colorimetric approach in the 0–2 nM range with a LOD of 0.29 nM (Figure 5E). Moreover, this biosensor has good selectivity for the detection of *C. difficile* RNase HII compared to RNase HII from other bacteria (Supplementary Figure S4B). The results demonstrate that the fluorescent approach agrees well with the colorimetric approach. The combination of fluorescent and colorimetric approaches can improve detection accuracy.

To demonstrate the practical application of the Pd-Ir nanocube-based biosensors for real samples, we spiked different concentrations of *C. difficile* RNase HII in tap water (from our lab) in the range of 0.5–2 nM and detected RNase HII by the fluorescent biosensors. As summarized in Supplementary Table S2, the recoveries for the four RNase HII spiked tap water samples were measured to be between 92.27 and 105.52%. The coefficient of variation (n = 6) was below 10.13% for all samples. The results imply that the performance of Pd-Ir nanocube-based biosensors was not compromised by tap water, suggesting the feasibility of this biosensor for real sample detection.

## Conclusion

In summary, a simple and surfactant-free method is developed here to synthesize Pd-Ir nanocubes with atomic Ir shells in an aqueous solution at room temperature. A long-standing issue with the coating of Ir on Pd nanocubes is addressed by simply adjusting the reaction kinetic with the concentrations of Na_3_IrCl_6_ and NaBH_4_. The Pd-Ir nanocubes have uniform particle size and excellent peroxidase-mimicking activity, due to the intrinsic electronic structure of Ir, electronic effect between Pd and Ir, and clean surface. Pd nanocubes with different sizes can be coated with atomic Ir layers using this approach. Due to its surfactant-free synthesis, Pd-Ir nanocubes can be conjugated with antibodies by physical adsorption for immunoassay, avoiding the tedious and complex chemical conjugation processes. Pd-Ir nanocubes can also be combined with fluorescent aptamers for the detection of *C. difficile* RNase HII by both fluorescence and colorimetric reporting. The method presented here makes a significant step forward towards the practical application of Pd-Ir nanocubes for biosensing.

## Data Availability

The original contributions presented in the study are included in the article/Supplementary Material, further inquiries can be directed to the corresponding author.
