# Supplementary material for: Facile Synthesis of Pd-Ir Nanocubes for Biosensing
Source: Front Chem. 2021 Nov 24;9:775220. doi: 10.3389/fchem.2021.775220 (PMC8651546; doi:10.3389/fchem.2021.775220)
Supplement: Supplementary file 1 [file DataSheet1.PDF]

Supplementary Material

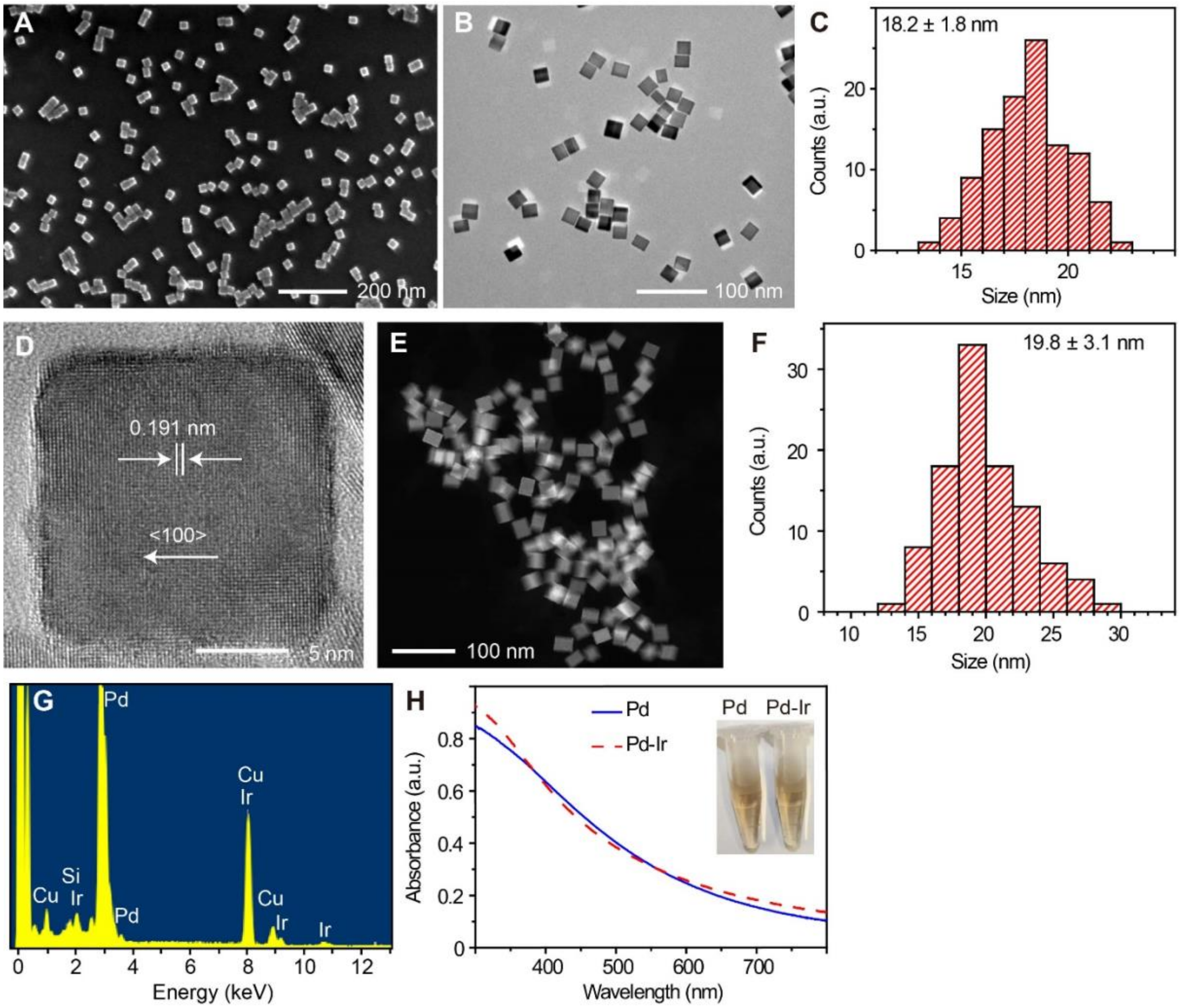

Figure S1. (A) The SEM image of Pd nanocubes. (B) The TEM image of Pd nanocubes. (C) Size distribution analysis of Pd nanocubes. (D) The HRTEM image of an individual Pd nanocube. (E) The HAADF-STEM image and (F) size distribution analysis of Pd-Ir nanocubes. (G) EDX analysis of Pd-Ir nanocubes. (H) UV-Vis absorption spectra (inset: pictures) of Pd nanocubes and Pd-Ir nanocubes.

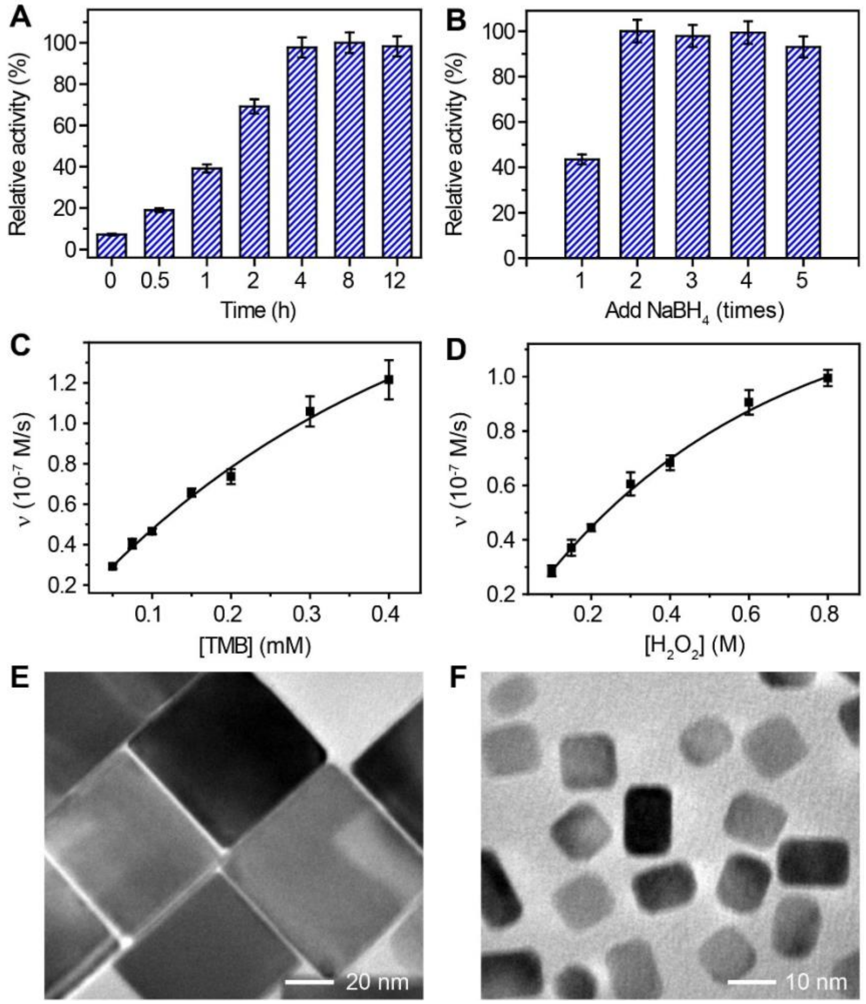

Figure S2. (A) Effect of reaction time on the catalytic activities of Pd-Ir nanocubes. (B) Effect of repetition times of $NaBH_4$ addition on the catalytic activities of Pd-Ir nanocubes. Plots of initial reaction velocity against (C) TMB or (D) H_2O_2 concentrations, reaction catalyzed by 18 nm Pd-Ir nanocubes. The TEM images of (E) 51 nm and (F) 7 nm Pd nanocubes.

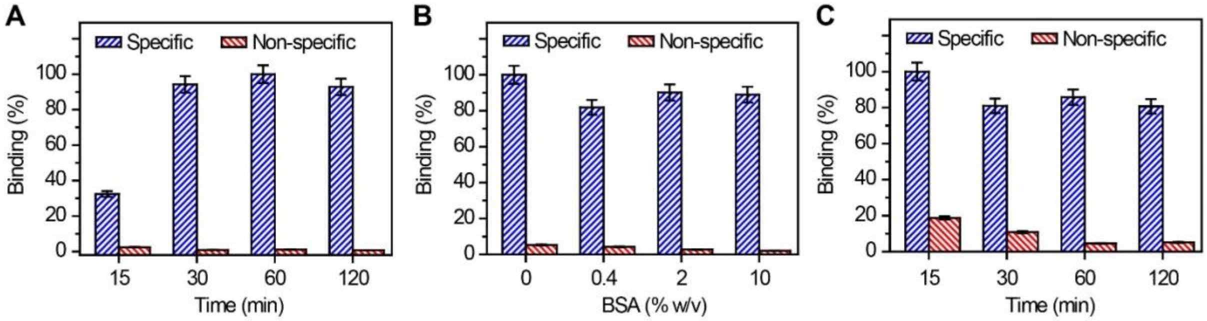

Figure S3. (A) Effect of antibody incubation times on specific binding of Pd-Ir nanocubes. Effect of (B) BSA concentrations and (C) BSA incubation time on non-specific binding of Pd-Ir nanocubes. Specific binding refers to conduct immunoassay using Pd-Ir nanocubes in the presence of PSA target (32 pg/mL), while non-specific binding in the absence of PSA target.

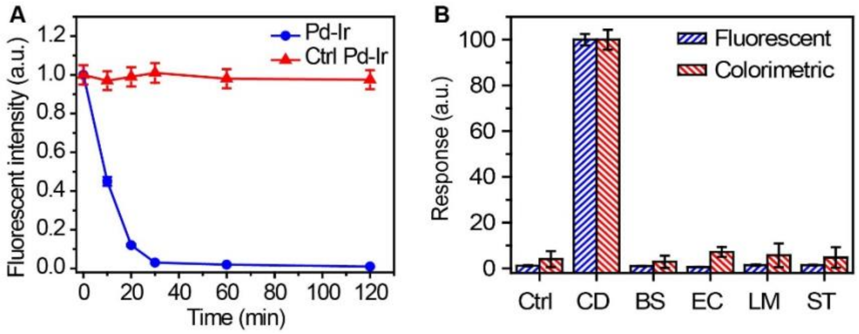

Figure S4. (A) Adsorption kinetic of fluorescein-labelled DNA on Pd-Ir nanocubes synthesized in this work (Pd-Ir) or synthesized according to literature (Ctrl Pd-Ir). (B) Selectivity of Pd-Ir nanocube-based biosensor for the detection of *C. difficile* RNase HII; CD: *C. difficile*, BS: *B. subtilis*, EC: *E. coli*, LM: *L. monocytogenes*, ST: *S. typhimurium*.

Table S1. Steady-state kinetic assays of Pd-Ir nanocubes for catalyzing the oxidation of TMB with H_2O_2.

Catalyst	E (M)	Substrate	K_m (mM)	V_{max} (M/s)	k_{cat} (s^{-1})	
Pd-Ir	6.88×10^{-14}	TMB	0.32	2.05×10^{-7}	2.91×10^{6}	This work
	6.88×10^{-14}	H_2O_2	520	1.64×10^{-7}	2.33×10^{6}	
Pd-Ir	3.4×10^{-14}	TMB	0.13	6.5×10^{-8}	1.9×10^{6}	reference[1]
	3.4×10^{-14}	H_2O_2	340	5.1×10^{-8}	1.5×10^{6}	
HRP	2.5×10^{-11}	TMB	0.43	1.0×10^{-7}	4.0×10^{3}	reference[2]
	2.5×10^{-11}	H_2O_2	3.7	8.7×10^{-8}	3.5×10^{3}	

Table S2. Recoveries of Pd-Ir nanocube and aptamer-based fluorescence biosensors for the detection of *C. difficile* RNase HII spiked in tap water.

Spiked RNase HII (nM)	Measured RNase HII (nM)	Coefficient of variations (%, n = 6)	Recovery (%)[a]
0.50	0.46	10.13	92.27
1.00	0.98	7.24	98.18
2.00	2.11	4.31	105.52
4.00	3.95	5.26	98.75

[a]: Recovery defined as the measured concentration of RNase HII divided by the concentration of RNase HII in original spiked tap water.
